# Development of an indirect ELISA based on a new specific lipoprotein LP53 for the detection of antibodies against *Mycoplasma synoviae*

**DOI:** 10.1186/s12917-025-04827-4

**Published:** 2025-05-31

**Authors:** Haoran Li, Zengjin Hu, Guijun Wang, Yu Wang, Shaohui Wang, Mingxing Tian, Yanqing Bao, Jingjing Qi, Shengqing Yu

**Affiliations:** 1 Shanghai Veterinary Research Institute, the Chinese Academy of Agricultural Sciences (CAAS), 518 Ziyue Road, Shanghai, 200241 P. R. China; 2 College of Animal Science and Technology, Anhui Agricultural University, No. 130, Changjiangxilu, Hefei, Anhui 230061 PR China; 3 College of Veterinary Medicine, Yangzhou University, No. 88 University South Road, Yangzhou, Jiangsu 225009 P. R. China

**Keywords:** *Mycoplasma synoviae*, LP53, Immunogenic lipoprotein, Indirect ELISA, Infection serum detection

## Abstract

**Background:**

*Mycoplasma synoviae* (MS) is considered to be one of the main mycoplasma pathogens of poultry, causing arthritis, airsacculitis, eggshell apex abnormalities and production drops in chickens and turkeys. Infection by MS usually results in considerable economic losses to the poultry industry worldwide. Therefore, it is essential to develop a highly sensitive and accurate diagnostic method in the livestock production.

**Results:**

The MSLP53 was predicted as a highly conserved and specific membrane associated lipoprotein of MS by bioinformatics analysis. The His-tagged MSLP53 (rMSLP53) protein was expressed and purified using *E. coli* expression system, and was confirmed by Western blotting to react with each MS-positive serum, but not react with positive sera against other avian pathogens, suggesting that the rMSLP53 had strong immunoreactivity and specificity. An rMSLP53-based indirect ELISA was developed, compared to IDEXX kit with a pool of 277 chicken sera samples, and showed high sensitivity (85.54%), specificity (89.19%) and coincidence rate (87.00%) within these two methods. When detecting the sera from experimentally infected chickens, the newly established rMSLP53-ELISA had certain advantages over the IDEXX kit, that is, it could identify the serum 3–18 weeks after infection as MS-positive serum, while IDEXX kit could only identify the serum 3–12 weeks after infection as positive serum.

**Conclusion:**

The MSLP53 protein is a specific immunogenic lipoprotein of MS, which was confirmed to be a promising antigen target for the detection of serum antibodies of MS. A rMSLP53-based indirect ELISA assay was successfully established in this study, showed high sensitivity, specificity and consistency with the commercial IDEXX kit, and could recognize the serum as MS-positive in a longer period after MS infection than IDEXX kit. This newly established rMSLP53-ELISA may be used as an effective detection method for MS serological monitoring and epidemiological investigation.

**Supplementary Information:**

The online version contains supplementary material available at 10.1186/s12917-025-04827-4.

## Background

Avian mycoplasmosis is caused by pathogens from genus *Mycoplasma* within the class of Mollicutes, which was first described as chronic respiratory disease caused by coccobacilliform bodies in 1936 [1]. Among a total of 25 known species of mycoplasma in poultry, primarily four species, *Mycoplasma gallisepticum* (MG), *Mycoplasma synoviae* (MS), *Mycoplasma meleagradis* (MM) and *Mycoplasma iowae* (MI) are considered to be pathogenic [2]. MS is considered to be one of the main pathogenicity of poultry, causing abnormal ities in the synovial membranes of joints and tendon sheaths, with eggshell apex abnormal ities and production drops [3]. Furthermore, it causes synergistic effects in producing diseases with other pathogens, such as Newcastle disease virus (NDV), Infectious bronchitis virus (IBV), Avian influenza virus (AIV) and *Escherichia coli* (*E. coli*) [4–6]. Thus, MS infections are a huge concern for poultry farmers and cause huge economic losses to poultry industry every year [7]. MS was first isolated in the United States in 1954, although it can be found in poultry throughout the world [8–11]. Due to MS having been traditionally considered as one of the most important avian mycoplasma species in commercial chickens from the clinical and economic point of view, it was listed and notifiable to the World Organization for Animal Health (OIE) [12, 13]. In recent years, along with the increasing development of the poultry industry, multi-aged breeder farms were widely infected with MS between 2010 and 2015 in China. MS infection is very common in China and should prompt further research to develop effective control and prevention strategies [14]. The sero-prevalence of MS has been observed much higher than that of MG, especially in countries with control and eradication programmes for the latter [15]. Therefore, the efficient, sensitive, and rapid diagnostic method of MS is urgently required to identify infected poultry to reduce the risk of transferring the infection to healthy poultry, as well as prioritize care and controls measures in geographical regions in which MS is highly prevalent.

Isolation and identification are the “gold standard” of mycoplasma diagnosis, but due to the difficulty of cultivation and long culture time, it is not suitable for rapid clinical diagnosis. Large-scale primary screening of flocks for MS infection typically uses serological diagnostic assays such as serum plate agglutination (SPA), enzyme linked immunosorbent assay (ELISA), and haemagglutination inhibition (HI) [2]. In both surveillance and epidemiological research, the serological detection has been widely utilized to identify infected and carrier animals [2]. While HI and SPA remain common diagnostic tools for MS, their reliance on whole-cell antigens or hemagglutination activity introduces notable limitations. HI assays are labor-intensive and exhibit limited sensitivity in subclinical infections, whereas SPA, despite rapid turnaround, suffers from subjective interpretation and cross-reactivity with non-MS mycoplasmas [16]. Numerous laboratories employ commercially available ELISA kits for screening or confirmation purpose. In general, ELISA tests exhibit slightly reduced sensitivity but greater specificity compared to SPA tests, while demonstrating higher sensitivity but lower specificity relative to HI tests [16–19]. Therefore, it is essential to screen out useful markers of MS infection for developing sensitive and specific serological tests. In MS, the membrane surface is abundant in lipoproteins, which play a critical role in adhesion to host cells and are considered key targets of the host immune response [20–22]. The membrane-associated lipoprotein MSPB, located at the amino-terminal end of the variable lipoprotein hemagglutinin (VlhA), has been recognized as an important immune response protein, and a highly specific and sensitive diagnostic antigen for detection of MS serum antibody [23, 24]. However, the MSPB contains a proline-rich repeat region, which is prone to insertion or deletion mutation and resulting in antigenic variation [25, 26]. This genetic instability may result in false-negative outcomes during clinical testing, significantly compromising diagnostic reliability. To overcome these challenges, there is an urgent demand for a novel diagnostic antigen that integrates strong immunoreactivity, exceptional specificity, high genetic stability, and compatibility with high-throughput platforms. In previous study, we screened an immunogenic protein MSLP53 by immunoproteomics with mass spectrometry analysis of MS membrane proteins (data not shown). Its remarkble intraspecific conservation significantly may reduce the risk of false-negative results due to strain-specific mutations, while its high interspecific specificity may minimize false-positive results by preventing cross-reactivity with other strains. The objective of this study is to develop and optimize an indirect ELISA assay utilizing the MSLP53 protein, thereby establishing a critical tool for the accurate monitoring and effective control of MS infections.

## Results

### Bioinformatic analysis of MSLP53

The MSLP53 protein contains 498 amino acids with an estimated molecular mass of 53.6 kDa and an isoelectric point of 6.81 using the Compute pI/Mw tool of ExPASy. It is speculated that MSLP53 is a membrane-associated lipoprotein because the MSLP53 protein was predicted containing a prokaryotic membrane lipoprotein lipid attachment site profile (PROKAR_LIPOPROTEIN) at the N-terminal by the Prosite tool. Homology alignment analysis by BLASTp against Non-redundant protein sequences (nr) database showed the similarities of LP53 between 26 different MS isolates (including MS WVU_1853_, MS NCTC10121, MS-H and MS 53, MS HB11, MS 86079-7NS, MS CH49, MS CH56, MS GX11-T, MS SH, MS 18DW, MS BS4S2, MS G3, MS A4, MS Zhejiang yqh71, MS Guangxi_zxm61, MS Guangxi_1mm64, MS GuangXi_wxm43, MS GuangXi_h12a15, MS Guangdong_tz159, MS JiangSu_xh51, MS JiangSu_gyw72, MS XiNan_ylk67, MS XiNan_wzy60, MS XiNan_zcs44, and MS Fujian_hzh45) were 97.79-100% under a query cover value of 100%, while the homology with other species was less than 26.34%.

### Expression of MSLP53 protein in *E. coli*

**Fig. 1:**
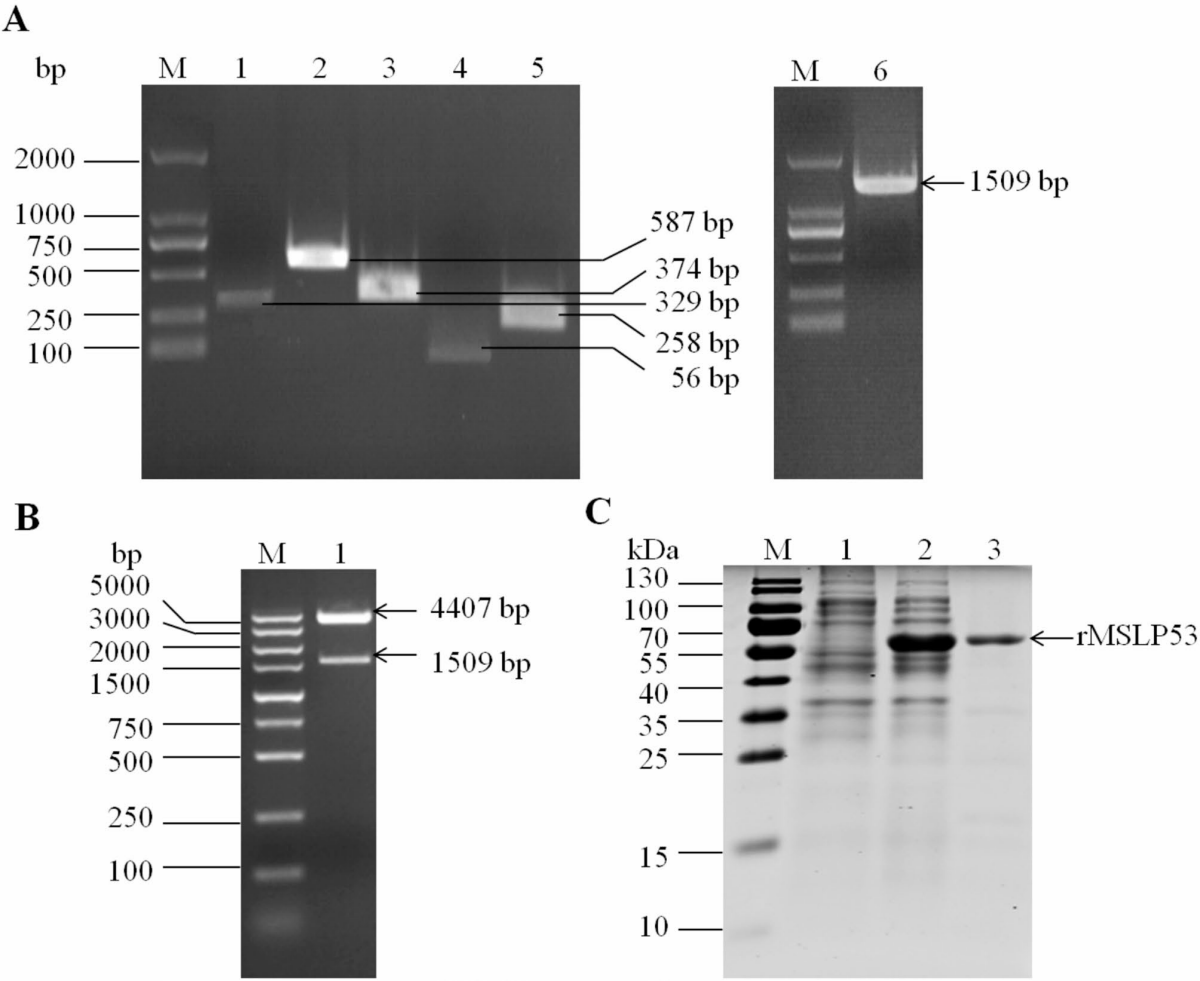
Amplification, recombinant plasmid construction, expression and purification of MSLP53. (**A**) Overlap PCR products for MS*lp53* gene. M: DL2 000 DNA marker; 1–5: PCR product with primers MS*lp53* 1 F/1R-5 F/5R; 6: full length of MS*lp53* gene produced by overlap PCR amplification. (**B**) Double enzyme digestion of recombinant expression plasmid. M: DL5 000 DNA marker; 1: the recombinant expression plasmid pCold I-MS*lp53* digested by *Bam*H I/*Eco*R I. (**C**) Expression and purification of recombinant MSLP53 protein. 1: IPTG-induced *E. coli* BL21 cells containing empty pCold I vector; 2: supernatants from IPTG induced *E. coli* BL21 containing pCold I-MS*lp53*; 3: purified rMSLP53 protein

The full length of MS*lp53* gene, which was modified (TGA to TGG) at four sites, was obtained from the MSWVU_1853_ genome with overlap PCR (Fig. 1A). It was then subcloned to pCold I vector (Fig. 1B) and transformed into *E. coli* BL21(DE3). After induction with β-D-1-thiogalactopyranoside (IPTG), the His-tagged MSLP53 protein was expressed in *E. coli* as a soluble protein and was purified successfully (Fig. 1C). The concentration of the purified rMSLP53 protein was about 0.6 mg/mL as determined by a BCA protein concentration detection kit.

### Immunoreactivity and specificity analysis of rMSLP53

**Fig. 2:**
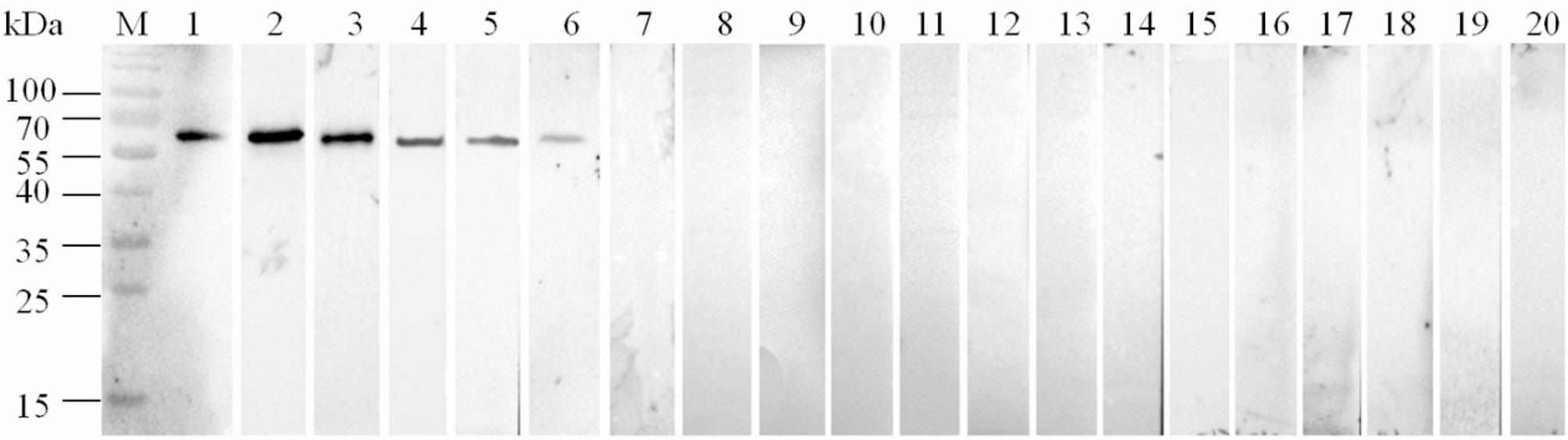
Immunoreactivity and specificity analysis of rMSLP53 with different chicken sera. The purified rMSLP53 protein was used as antigen to react with chicken positive sera against different MS isolates, MG-positive chicken sera and positive sera against other avian pathogens. M: protein marker; Lane1-6: the standard MS-positive chicken serum (positive control) and positive chicken sera of different MS isolates (MS WVU_1853_, JS1, SD1, SH1 and HB1); Lane7-13: standard MG-positive chicken serum and different MG-positive chicken sera (MG R_low_, 013, 08, FBH, SGN, SS); Lane14-19: positive sera against other avian pathogens including (MI, SPG, *E. coli* O1/O2/O78, NDV, IBV and IBDV; Lane20: MS-negative serum (negative control)

Immunoblot assays were performed to analyze the immunoreactivity and specificity of rMSLP53. The purified rMSLP53 protein was used as antigen to react with the standard MS-positive chicken serum and chicken positive sera against different MS isolates (MS WVU_1853_, JS1, SD1, SH1 and HB1), the results indicated that the rMSLP53 appeared to have a specific reaction band with each MS-positive serum (Fig. 2, lanes 1–6), but no band with standard MG-positive chicken serum and different MG-positive chicken sera (Fig. 2, lanes 7–13), positive sera against other avian pathogens including MI, SPG, *E. coli* O1/O2/O78, NDV, IBV and IBDV (Fig. 2, lanes 14–19), or MS-negative serum (Fig. 2, lane 20). These findings suggested that the rMSLP53 had strong immunoreactivity and specificity.

### Determination of optimal conditions for indirect ELISA

A square matrix titration test was used in this study to search for the optimal reaction conditions. The reaction conditions corresponding to the highest P/N values are generally considered to be the optimal conditions. As shown in Table 1, due to the highest P/N value, the optimal concentration of the rMSLP53 protein as the coating antigen was 0.63 µg/mL, and the optimal dilution of chicken serum was 1:500. The other reaction conditions were optimized by varying a single parameter at a time. The optimal dilution of HRP conjugated goat anti-chicken IgY antibody was 1:20 000 (Table 2).

**Table 1:** Optimization of rMSLP53 protein coating amount and serum dilution

Serum dilutions	Serum	rMSLP53 protein coating concentration (µg/mL)							
		10.00	5.00	2.50	1.25	0.63	0.31	0.16	0.08
	Positive	1.742	1.647	1.576	1.471	1.334	1.141	0.846	0.639
1: 200	Negative	0.498	0.478	0.454	0.318	0.219	0.145	0.138	0.123
	P/N	3.498	3.446	3.471	4.626	6.091	7.869	6.130	5.195
	Positive	1.444	1.332	1.284	1.202	1.274	0.929	0.685	0.471
1: 500	Negative	0.364	0.318	0.264	0.208	0.129	0.124	0.114	0.124
	P/N	3.967	4.189	4.864	5.779	**9.876**	7.492	6.009	3.798

Note: The values in the table represent the OD_450nm_ values for positive and negative serum samples. The P/N represents the ratio of positive to negative OD_450nm_ values

**Table 2:** Optimization of indirect ELISA conditions of enzyme-labeled antibody

	Dilution of enzyme-labeled antibody			
	1:10 000	1: 20 000	1: 40 000	1: 60 000
Positive (OD_450nm_)	1.340	1.249	0.689	0.521
Negative (OD_450nm_)	0.149	0.125	0.103	0.093
P/N	7.929	**9.992**	6.683	5.602

Note: The dilution of HRP conjugated goat anti-chicken IgY antibody was optimized under the conditions of 0.63 µg/mL rMSLP53 as the coating antigen and a serum dilution ratio of 1:500. The values in the table represent the OD_450nm_ values for positive and negative serum samples. The P/N represents the ratio of positive to negative OD_450nm_ values

### Application of the indirect ELISA assay in clinical samples

**Fig. 3:**
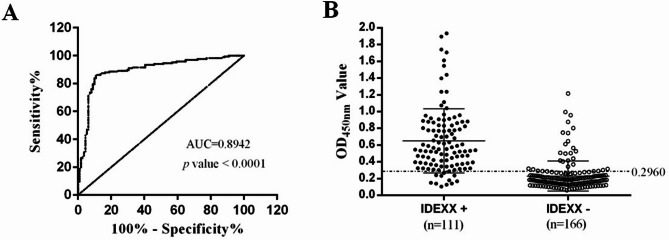
Determination of the cut-off value and analysis of sensitivity and specificity based on ROC curves. (**A**) ROC analysis used to determine area under the curve values (AUC = 0.8942, *p* value < 0.0001) for the indirect ELISA method using IDEXX kit test as the reference. The optimal cut-off value was determined as the point on the ROC curve closest to the top-left corner. Based on the ROC curve analysis, the optimized sensitivity and specificity were 85.54% and 89.19%, respectively, with the cut-off value set at OD_450nm_ = 0.2960. (**B**) Distributions of OD_450nm_ values for MS-positive and MS-negative serum samples using the indirect ELISA method. A total of 111 IDEXX kit test positive (IDEXXT+) and 166 IDEXX kit test negative (IDEXXT-) sera were evaluated by the indirect ELISA. The dashed line represents the cut-off value for this indirect ELISA based on ROC analysis. The values above the dashed line are considered as MS-positive and those below as MS-negative according to cut-off value for this indirect ELISA

MS antibody test kit (IDEXX, USA) identified 111 clinical MS-positive serum and 166 clinical negative serum samples. The OD_450nm_ values of these clinical serum samples were observed by the optimal indirect ELISA method as described above. As shown in Fig. 3A, the area under the ROC curve (AUC) was calculated to be 0.8942 with a statistically significant *p* value of < 0.0001, indicating that the rMSLP53-indirect ELISA has satisfactory diagnostic performance. Based on the ROC curve analysis (Fig. 3B), an optimal cut-off value of OD_450nm_ = 0.2960 was identified, corresponding to the point closest to the top-left corner of the curve. This value maximizes Youden’s Index (sensitivity + specificity − 1), yielding a sensitivity of 85.54% and a specificity of 89.19%. The IDEXX kit and the rMSLP53-indirect ELISA together tested a total of 99 positive samples and 142 negative serum samples (Table 3). Thus, the positive and negative coincidence rates of the rMSLP53-indirect ELISA, compared to the IDEXX kit, are 89.19% and 85.54%, respectively, and the total coincidence rate of the two methods is 87.00%.

**Table 3:** Clinical Sera results from the indirect ELISA and IDEXX kit test

		IDEXX kit		Total
		Positive	Negative	
rMSLP53-based indirect ELISA	Positive	99 (89.19%)	24	123
	Negative	12	142 (85.54%)	154
	Total	111	166	277

Note: The percentages indicate the positive and negative coincidence rates of the rMSLP53-indirect ELISA compared to the IDEXX kit

### Application of the indirect ELISA assay in MS-infected chicken sera

**Fig. 4:**
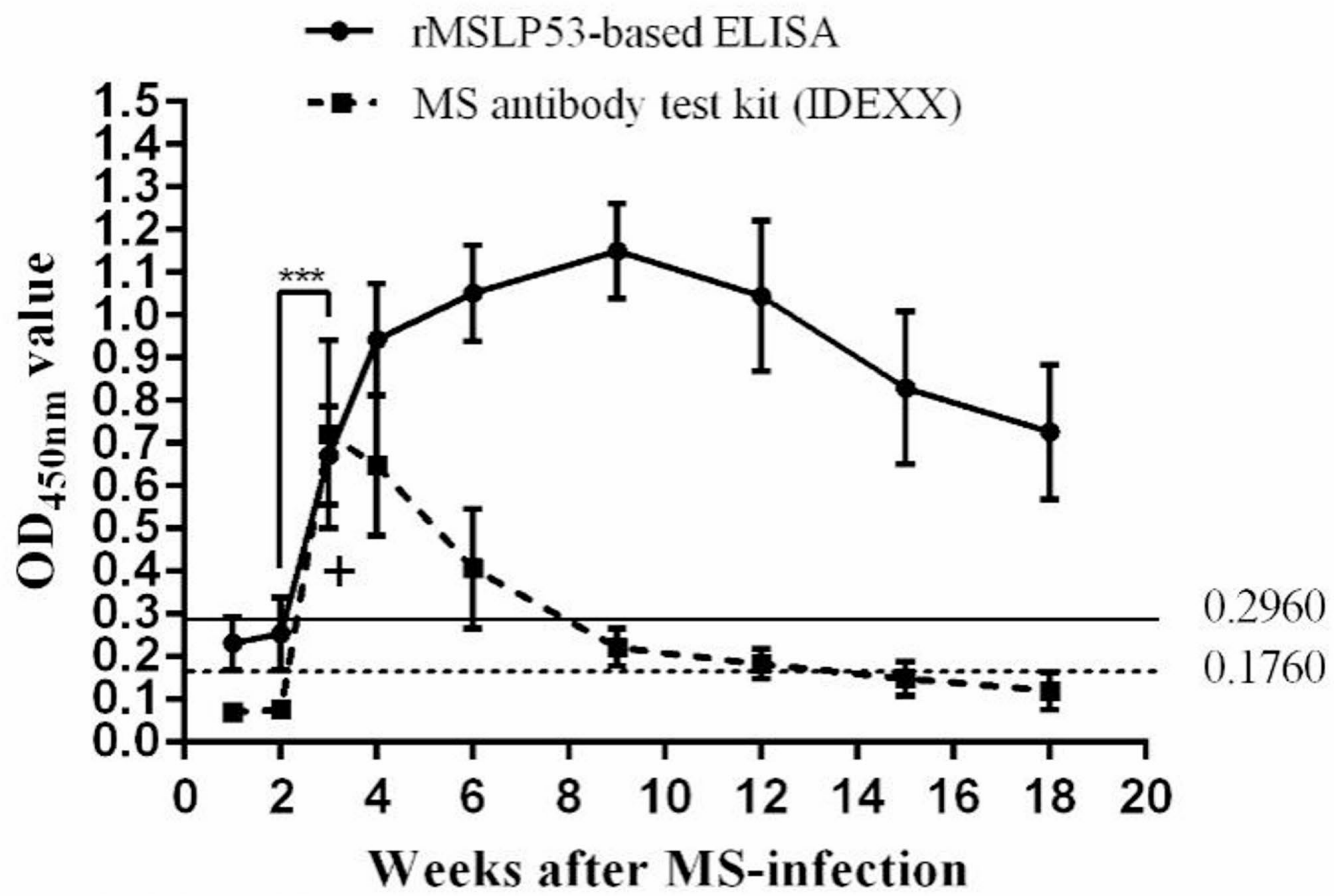
Detection of MS antibody levels in MS-infected chicken sera using rMSLP53-based ELISA. Sera from eight MS-infected chickens were subjected to the established indirect rMSLP53-based ELISA assay and compared with the MS antibody test IDEXX kit. The sera were collected at 1, 2, 3, 4, 6, 9, 12, 15, 18 weeks (^*^: *p* < 0.05; ^**^: *p* < 0.01; ^***^: *p* < 0.001). The cut-off value of rMSLP53-based ELISA was 0.2960 (horizontal straight line), which of the IDEXX kit was 0.1760 (horizontal dashed line)

Changes in MS antibody levels in antisera from eight MS-infected chickens were detected using both the rMSLP53-based ELISA assay and the IDEXX kit. As shown in Fig. 4, the antibodies were not detected in the first two weeks, but increased significantly in the third week for both two methods (*p* < 0.001). For the rMSLP53-based ELISA assay, the antibody level against MS showed a stable increase from the third week to the 9th week. After the 9th week, the antibody level decreased, but remained at a high level after 18th week. However, for the IDEXX kit, the sera antibodies against MS began to decrease after the third week, and no positive value was detected after 15th week.

### Reactivity, specificity and sensitivity of the rMSLP53-based ELISA

**Fig. 5:**
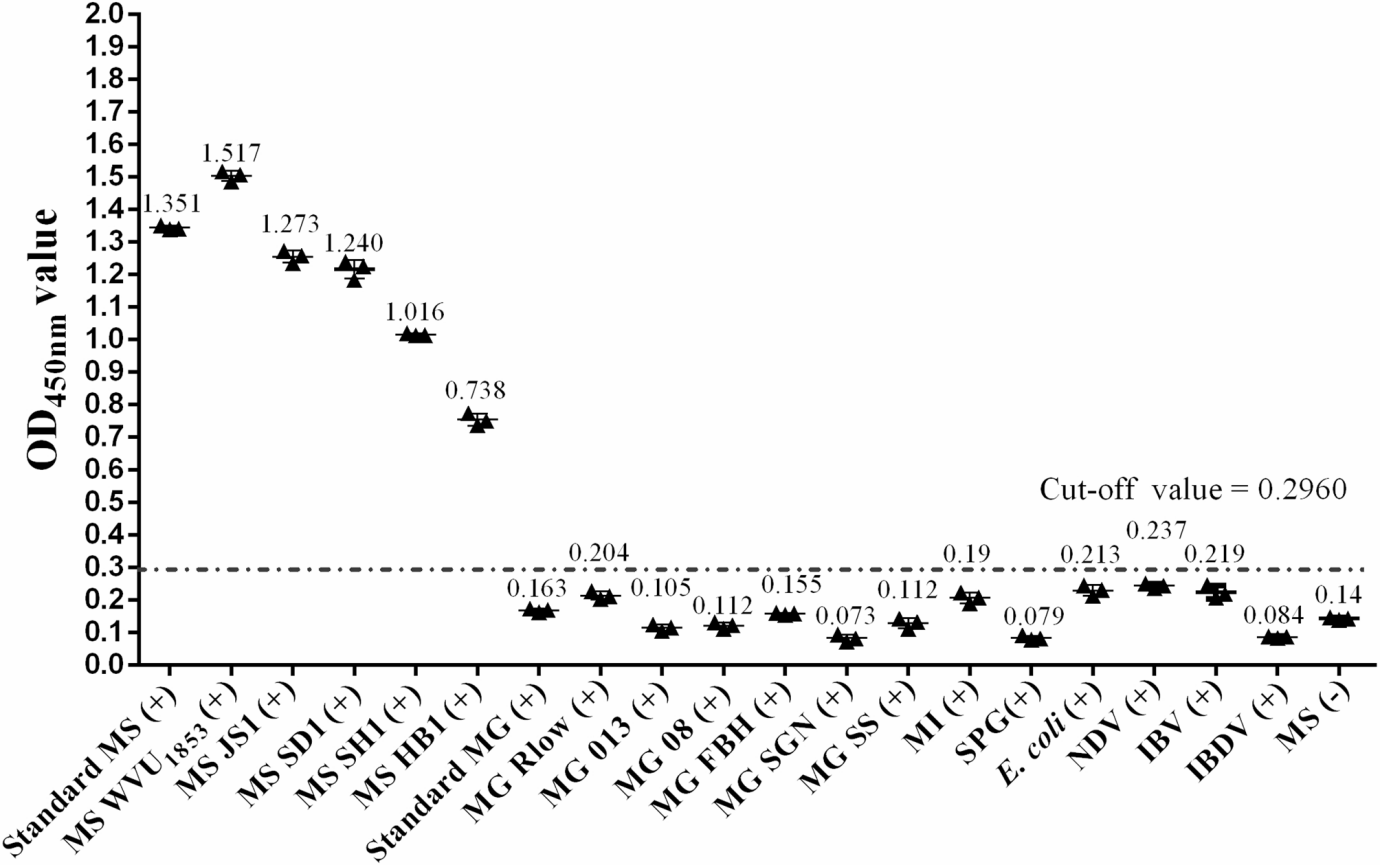
Reactivity and specificity analysis of the indirect ELISA. Positive chicken sera for common pathogens in poultry, including the standard MS-positive chicken serum (control), different MS isolates, different MG strains, MI, SPG, *E. coli* O1/O2/O78, NDV, IBV and IBDV were detected by the indirect ELISA and the OD_450nm_ values were recorded. The mean OD450nm values for each serum were shown directly above their corresponding data points in the figure. The OD_450nm_ values of standard MS-negative serum and the positive serum for 13 other pathogens were below the cut-off value (0.2960) except those of MS-positive sera

To investigate the reactivity and specificity of the indirect ELISA, chicken sera that were positive for MS different isolates, MG different strains, MI, SPG, *E. coli* O1/O2/O78, NDV, IBV and IBDV were used as the samples and followed by the established procedures. The results showed that when the indirect ELISA reacted with different MS-positive sera, the OD_450nm_ values were at a high level (greater than 0.7), but when reacted with positive sera against other avian pathogens, the OD_450nm_ values were less than 0.2960 (cut-off value determined by ROC analysis), indicating that the established indirect ELISA had excellent reactivity and specificity (Fig. 5).

The indirect ELISA detected five MS-infected serum samples in comparison with the IDEXX kit. The results confirmed that the sensitivity of the indirect ELISA was significantly better than that of the IDEXX kit (Table 4).

## Discussion

Avian mycoplasmosis mainly caused by MG and MS is an economically important disease of poultry industry [27]. MG and MS cause persistent infection in chickens, which have been in long-term clinical or subclinical state, triggering immunosuppression and other important infectious diseases such as NDV, AIV, and IBV [16]. Eradication is the most important control measure for MG and MS infections in poultry. For eradication of vertically transmitted agents, early detection of new infections is essential [28]. Many countries and integrations are involved in monitoring programmes to control both mycoplasma species. The epidemiology and harmfulness of MS in the poultry industry are becoming increasingly apparent, necessitating the development of an effective detection technology to prevent and control the spread of MS [29, 30]. At present, MS infection is often diagnosed using SPA, HI, or ELISA. Diagnosis of avian mycoplasmosis infections in poultry breeder flocks is often performed in the absence of overt clinical signs, and screening for infection is usually based on a presumptive test performed by SPA test and a confirmative test accomplished by ELISA [31]. SPA is highly efficient in detecting the earliest immune response of immunoglobulin M (IgM) to mycoplasma infection [31]. Although the SPA test is rapid, highly sensitive, and relatively inexpensive, it is prone to false positive results due to its tendency to cross-react with other sera such as MG, MI, and other sera, which can affect the accuracy of the diagnosis [31]. HI is a specific serological detection method and does not cause cross reaction between MG- and MS- positive serum. However, due to strict requirements for antigen preparation and storage, as well as complex operations and poor sensitivity, its use is limited to some extent. By comparison, ELISA can simultaneously detect a large number of samples with higher specificity, sensitivity, and accuracy, which is relatively stable and currently the most widely used method [32, 33]. In mycoplasma, a large amount of lipoproteins were exposed on the surface, which can be used as antigen to induce host immune response or play a toxic role [34–38]. In our early study, the immuno-proteomics analysis of MS membrane protein was conducted combining with mass spectrometry sequencing, the lipoprotein MSLP53 was found to be an immunogenic protein (data not shown). The MSLP53 protein is predicted to be a membrane-associated lipoprotein because the N-terminal of the MSLP53 protein contains a prokaryotic membrane lipoprotein binding domain. Due to the homology of MSLP53 protein sequence between 26 different MS isolates reaching 97.79-100%, while the homology with other species was less than 26.34%, the MSLP53 was considered a highly conserved and specific protein of MS. Later, the western blot analysis showed the purified rMSLP53 protein displayed a specific immune reaction with the positive sera of various isolates of MS and not with those of other avian diseases, indicating it is appropriate as an antigen for detecting specific antibodies against MS.

Here we established an indirect ELISA detection method based on rMSLP53 protein as a coated antigen. The rMSLP53-ELISA method was evaluated using 277 clinical serum samples from 3 poultry farms in different areas of China (including Shanghai, Shandong and Guangdong), and was then compared with the IDEXX kit. The sensitivity and specificity were detected at 85.54% and 89.19%, respectively, and the total coincidence rate of the two methods is 87.00%, which confirmed the application potential of rMSLP53-ELISA for MS antibody detection. In addition, the rMSLP53-based ELISA revealed that chickens infected by MS could produce high levels of antibodies against MSLP53 from 3 to 18 weeks after infection. However, the IDEXX testing method could only detect positive serum from 3 to 12 weeks after MS infection. These results suggest that the rMSLP53-based ELISA assay has certain advantages over the IDEXX kit in detection of MS-infected sera, because it can detect the positive value for a longer time after infection than the IDEXX kit.

This study is the first to use LP53 protein as a coating antigen to establish an indirect ELISA method for detecting MS serum antibodies and assess its potential application in clinical sera, providing a reference approach for the diagnosis and epidemiological investigation of MS.

## Conclusion

In conclusion, we identified a lipoprotein MSLP53 with strong intraspecific conservation and interspecific specificity that could effectively recognize MS-positive serum. This newly established rMSLP53-ELISA showed high sensitivity, specificity and consistency with the commercial IDEXX kit, and could recognize the serum as MS-positive in a longer period after MS infection compared with IDEXX kit, which may be used as an effective detection method for MS serological monitoring and epidemiological investigation.

## Materials and methods

### Experimental strains and serum

The MS strain WVU_1853_ was purchased from the China Veterinary Culture Collection Center (CVCC). The positive chicken sera against different MS clinical isolates (including MS strain WVU_1853_, JS1, SD1, SH1 and HB1), MG strains (MG R_low_, 013, 08, FBH, SGN, SS), MI 695, or *E. coli* O1/O2/O78 were prepared and preserved in our lab [20]. The standard MS/MG-positive, *Salmonella pullorum/gallinarum* (SPG)-positive, NDV-positive, IBV-positive chicken sera or Infectious bursal disease virus (IBDV)-positive, and standard negative chicken serum, were all obtained from the CVCC. A total of 277 clinical serum samples were collected from poultry farms in three different provinces of China, including 126 from Shanghai, 78 from Shandong and 73 from Guangdong.

### Bioinformatics analysis

The amino acid and nucleotide sequences of MSLP53 (VY93_02170) protein from MSWVU_1853_ (GenBank accession no: CP011096.1) were retrieved from the National Center for Biotechnology Information (NCBI) database and were used to conduct online BLASTp searches (https://blast.ncbi.nlm.nih.gov/Blast.cgi) against non-redundant protein sequences (nr) database. The theoretical isoelectric point (pI) and molecular weight (Mw) of the MSLP53 protein were calculated by Compute pI/Mw tool in Expasy (https://web.expasy.org/compute_pi/). The functional domains analysis of MSLP53 was using Prosite online software (https://prosite.expasy.org/cgi-bin/prosite/).

### Cloning and expression

The MS strain WVU_1853_ was obtained from the China Veterinary Culture Collection Center (CVCC, Beijing, China) and was grown in Modified Frey medium (BD, USA) supplemented with 0.01% NAD (Roche, Shanghai, China) and 10% porcine serum (Gibco, Carlsbad, CA, USA) at 37 °C in an atmosphere of 5% CO_2_. The *lp53* gene of MS (MS*lp53*) was cloned from the MSWVU_1853_ genome DNA with overlapping PCR. To circumvent the specific translational barrier of the TGA codon of mycoplasma in *E. coli*, the site-directed mutagenesis was conducted by overlapping PCR [39] to alter the TGA codon into TGG with 5 pairs of primer (Table 5). The full-length of MS*lp53* gene with the TGA to TGG modification at four sites was cloned into pCold I to generate the recombinant plasmid pCold I-MS*lp53*. The plasmid pCold I-MS*lp53* was transformed into *E. coli* strain BL21 (DE3), and the recombinant MSLP53 (rMSLP53) protein was then expressed with 0.5 mM IPTG induction at 16 °C, 110 rpm for 24 h. At last, the rMSLP53 protein was purified with BeaverBeads™ His protein purification kit (Beaver, Suzhou, China), and was analyzed with 10% sodium dodecyl sulfate polyacrylamide gel electrophoresis (SDS-PAGE) and coomassie brilliant blue staining.

**Table 4:** Comparison of sera titers detected by two methods

Serum	rMSLP53-based ELISA	IDEXX kit
1	1: 25 600	1: 800
2	1: 102 400	1:3200
3	1: 25 600	1:400
4	1: 6 400	1:1600
5	1: 25 600	1:1600

**Table 5:** Primers used for PCR amplification of *MSlp53* gene

Primers	Sequence (5´→3´)	sizes
*MSlp53* 1 F	*GGATCC*ATGAAAAAATTTGAATTTTTACTAC	329 bp
*MSlp53* 1R	GCACC**C**CATTTACCCGT**C**CA	
*MSlp53* 2 F	TG**G**ACGGGTAAATG**G**GGTGC	587 bp
*MSlp53* 2R	GATGCAGG**C**CATTTGTCGTATTTT	
*MSlp53* 3 F	AATACGACAAATG**G**CCTGCATCTAC	374 bp
*MSlp53* 3R	CGTTAAGTGC**C**CATTG**C**CAGC	
*MSlp53* 4 F	TTAAGCTG**G**CAATG**G**GCACTTAAC	56 bp
*MSlp53* 4R	GATCTTTAGCAGGGTTTGC**C**CATAA	
*MSlp53* 5 F	TTATTATG**G**GCAAACCCTGCTAAAG	258 bp
*MSlp53* 5R	*GAATTC*CTATTTTTTAGTTGCTGCAAGC	

Note: the restriction enzyme sites of *Bam*H I (GGATCC) and *Eco*R I (GAATTC) were underlined; the mutated nucleotides were in bold

### Immunoblot analysis of rMSLP53

The immunoreactivity and specificity analysis of rMSLP53 were evaluated by immunoblot assays. A total of 6.5 µg of purified rMSLP53 protein was divided into 13 portions and were subjected to 10% SDS-PAGE at a dose of 0.5 µg/lane. The 13 SDS-PAGE gels were transferred to NC membranes and blocked with 5% (w/v) no-fat milk in PBST (PBS buffer containing 0.05% Tween-20) at 4 °C overnight. After that, the membranes were individually incubated with positive chicken sera (1:500 diluted in PBST) of different avian pathogens at 37 °C for 1 h on a plate shaker. To assess the immunoreactivity of MSLP53 protein, the positive chicken sera against different MS strains (MS WVU_1853_, JS1, SD1, SH1 and HB1) were used. For specificity analysis, the positive chicken sera against different MG strains (MG R_low_, 013, 08, FBH, SGN, SS), standard MG-positive chicken serum, MI, SPG-positive, *Escherichia coli*O1/O2/O78-positive, NDV-positive, IBV-positive and IBDV-positive sera were used. The standard MS-positive and MS-negative chicken sera were used as positive and negative control respectively. Then the membranes were washed three times with PBST and incubated with HRP-conjugated goat anti-chicken IgY antibody (1:20 000; Abcam, UK) at room temperature at 37 °C for 30 min. After washing four times with PBST, the membranes were stained with an ECL chromogenic kit (Thermo, USA) and scanned using a multifunctional imaging system (Tanon 5200, China).

### Establishment of an indirect ELISA assay

Conventional indirect ELISA was performed as published by Wang et al. [36] with some modifications. The 96-wells ELISA plates (Corning, USA) were coated with rMSLP53 protein in 100 µL of 0.05 M carbonate-bicarbonate buffer (pH = 9.6) at 37°C for 2 h. The plates were then washed three times with PBST and blocked with 5% (w/v) no-fat milk in PBST (200 µL/well) for 2 h at 37°C. After washing three times, the plates were incubated with chicken sera to be checked (diluted in PBST, 100 µL/well) at 37°C for 1.5 h. After four washes, HRP-goat anti-chicken IgY antibody (diluted in PBST; Abcam) was added to the plates and incubated at 37°C for 1 h. Plates were thoroughly washed and reacted with 100 µL of 3,3’,5,5’-Tetramethyl benzidine (TMB) substrate solution at 37 °C for 15 min in complete darkness. The color reaction was stopped by the addition of 50 µL of 2 M H_2_SO_4_ to each well. Finally, the OD_450nm_ values were measured and recorded immediately using a multi-mode microplate reader (Synergy H1; BioTek, USA).

A checkerboard titration was performed on 96-well ELISA microplates to optimize the conditions for detection based on the method mentioned above. To determine the best concentration of the coating protein, rMSLP53 was diluted to the following concentrations: 10.00, 5.00, 2.50, 1.25, 0.63, 0.31, and 0.16 and 0.08 µg/mL. The standard MS-positive and MS-negative serum were diluted 1:200 and 1:500. The HRP-goat anti-chicken IgY antibody (100 µL/well) was optimized with different dilutions (1:10 000, 1: 20 000, 1:40 000, or 1:60 000) by PBST. The OD_450nm_ value of standard MS-positive value (P) was around 1.0, and the standard negative value (N) was less than 0.2, resulting in a maximum ratio of P and N (P/N) of no less than 2.1, which was regarded to be the best reaction circumstances [40].

### Clinical serum samples test

A total of 277 clinical serum samples from poultry farms in three different provinces in China (including Shanghai, Shandong and Guangdong) were examined in parallel, comparing a MS antibody test kit (IDEXX) and the newly developed rMSLP53-based ELISA. The cut-off value indicative of optimal sensitivity and specificity of the rMSLP53-based indirect ELISA were evaluated by receiver operating characteristic (ROC) analysis using GraphPad Prism 6.

### Detection of MS antibody levels change in MS-infected chicken

The rMSLP53-based ELISA assay was also used to evaluate the development of antibodies in chickens after infected by MS. A total of 16 3-week-old SPF chickens were purchased from Zhejiang Hengda Agricultural Development Co., Ltd., China, and divided into two groups. The MS WVU_1853_ were cultivated to late logarithmic growth stage, collected by centrifugation at 8000 g for 10 min, resuspended with PBS, and then were used for tracheal challenge to eight SPF chickens at the dose of 1 × 10^6^ color change units (CCUs) per chicken. The remaining 8 SPF chickens were tracheal challenged with equal volume PBS. After challenge, the chicken sera were collected at 1, 2, 3, 4, 6, 9, 12, 15, 18 weeks and then preserved at -40 ℃. The MS-infected sera were detected by rMSLP53-based ELISA and compared with the IDEXX kit.

### Specificity and sensitivity test

The rMSLP53-based ELISA method described above was used to simultaneously detect the OD_450nm_ values of chicken sera (1:500) positive for different MS isolates (WVU_1853_, JS1, SD1, SH1, and HB1), different MG isolates (R_low_, 013, 08, FBH, SGN, SS), and other avian pathogens including MI, SPG, *E.coli* O1/O2/O78, NDV, IBV, and IBDV. The standard MS-positive and MS-negative sera (1:500) were served as the positive and negative controls. Five MS-infected serum samples were serially diluted in a 2-fold series from 1: 50 to 1: 102,400 and conducted by the indirect ELISA procedures to determine sensitivity. The results were compared with those from IDEXX MS antibody test kit.

### Statistical analysis

The optimal cut-off value for the rMSLP53-based indirect ELISA, balancing sensitivity and specificity, was determined through ROC analysis using GraphPad Prism 6. The AUC serves as an indicator of a test’s capacity to accurately differentiate between individual samples. An AUC of 1.0 indicates perfect diagnostic performance, while a value of ≤ 0.5 suggests that the test lacks meaningful diagnostic utility [41]. The optimal cut-off value was identified as the point on the ROC curve nearest to the top-left corner, as this point equally maximizes sensitivity and specificity, thus maximizing the Youden’ Index (calculated as sensitivity + specificity − 1) [42].

## Electronic supplementary material

Below is the link to the electronic supplementary material.

Supplementary Material 1

## Data Availability

No datasets were generated or analysed during the current study.

## Abbreviations

ELISA: Enzyme-linked immunosorbent assay
MS: *Mycoplasma synoviae*
rMSLP53: Recombinant LP53 from MS
MG: *Mycoplasma gallisepticum*
MI: *Mycoplasma iowae*
E. coli: *Escherichia coli*
SPG: *Salmonella pullorum*/*gallinarum*
NDV: Newcastle disease virus
IBV: Avian infectious bronchitis virus
IBDV: Infectious bursal disease virus
SPA: Serum plate agglutination
HI: Haemagglutination inhibition
pI: Isoelectric point
Mw: Molecular weight
PCR: Polymerase chain reaction
IPTG: β-D-1-thiogalactopyranoside
ROC: Operating characteristic
AUC: Area under the ROC curve
SDS-PAGE: Sodium dodecyl sulfate polyacrylamide gel electrophoresis
HRP: Horseradish peroxidase
ECL: Enhanced chemiluminescence
TMB: 3,3’,5,5’-Tetramethyl benzidine
NC: Nitrocellulose filter
SPF: Specific pathogen free
CCU: Color change unit
PBS: Phosphate buffer saline
OD: Optical density
NAD: Nicotinamide adenine dinucleotide

## References

[1] Nelson JB. Studies on an uncomplicated Coryza of the domestic fowl: Vi. Coccobacilliform bodies in birds infected with the Coryza of slow onset. J Exp Med. 1936;63(4):515–22. 19870486 10.1084/jem.63.4.515 PMC2133352

[2] Yadav JP, Tomar P, Singh Y, Khurana SK. Insights on *Mycoplasma gallisepticum* and *Mycoplasma synoviae* infection in poultry: a systematic review. Anim Biotechnol. 2022;33(7):1711–20. 33840372 10.1080/10495398.2021.1908316

[3] Kleven SH. Control of avian mycoplasma infections in commercial poultry. Avian Dis. 2008;52(3):367–74. 18939621 10.1637/8323-041808-Review.1

[4] Springer WT, Luskus C, Pourciau SS. Infectious bronchitis and mixed infections of *Mycoplasma synoviae* and *Escherichia coli* in gnotobiotic chickens. I. Synergistic role in the airsacculitis syndrome. Infect Immun. 1974;10(3):578–89. 4609905 10.1128/iai.10.3.578-589.1974 PMC422992

[5] Sid H, Benachour K, Rautenschlein S. Co-infection with multiple respiratory pathogens contributes to increased mortality rates in Algerian poultry flocks. Avian Dis. 2015;59(3):440–6. 26478165 10.1637/11063-031615-Case.1

[6] Feberwee A, Morrow CJ, Ghorashi SA, Noormohammadi AH, Landman WJ. Effect of a live *Mycoplasma synoviae* vaccine on the production of eggshell apex abnormalities induced by a *M. synoviae* infection preceded by an infection with infectious bronchitis virus D1466. Avian Pathol. 2009;38(5):333–40. 19937520 10.1080/03079450903183652

[7] Ter Veen C, de Wit JJ, Feberwee A. Relative contribution of vertical, within-farm and between-farm transmission of *Mycoplasma synoviae* in layer pullet flocks. Avian Pathol. 2020;49(1):56–61. 31509002 10.1080/03079457.2019.1664725

[8] Michiels T, Welby S, Vanrobaeys M, Quinet C, Rouffaer L, Lens L, et al. Prevalence of *Mycoplasma gallisepticum* and *Mycoplasma synoviae* in commercial poultry, racing pigeons and wild birds in Belgium. Avian Pathol. 2016;45(2):244–52. 26814376 10.1080/03079457.2016.1145354

[9] Feberwee A, de Vries TS, Landman WJ. Seroprevalence of *Mycoplasma synoviae* in Dutch commercial poultry farms. Avian Pathol. 2008;37(6):629–33. 19023760 10.1080/03079450802484987

[10] Messa Júnior A, Taunde P, Zandamela AF, Junior AP, Chilundo A, Costa R, Bila CG. Serological screening suggests extensive presence of *Mycoplasma gallisepticum* and *Mycoplasma synoviae* in backyard chickens in Southern Mozambique. J Vet Med. 2017;2017:2743187. 10.1155/2017/2743187 PMC5294219 28243629

[11] Buim MR, Mettifogo E, Timenetsky J, Kleven S, Ferreira A. Epidemiological survey on *Mycoplasma gallisepticum* and *M. synoviae* by multiplex PCR in commercial poultry. Pesquisa Veterinária Brasileira. 2009;29(7):552–6.

[12] World Organization for Animal Health (OIE). Avian mycoplasmosis (*Mycoplasma gallisepticum*, *M. synoviae*). In Manual of diagnostic tests and vaccines for terrestrial animals 2019;II:844–59. Paris, France: OIE.

[13] Chaidez-Ibarra MA, Velazquez DZ, Enriquez-Verdugo I, Castro Del Campo N, Rodriguez-Gaxiola MA, Montero-Pardo A, et al. Pooled molecular occurrence of *Mycoplasma gallisepticum* and *Mycoplasma synoviae* in poultry: A systematic review and meta-analysis. Transbound Emerg Dis. 2021;65(5):2499–511. 10.1111/tbed.14302 34427387

[14] Xue J, Xu MY, Ma ZJ, Zhao J, Jin N, Zhang GZ. Serological investigation of *Mycoplasma synoviae* infection in China from 2010 to 2015. Poult Sci. 2017;96(9):3109–12. 28637299 10.3382/ps/pex134

[15] Landman WJ. Is *Mycoplasma synoviae* outrunning *Mycoplasma gallisepticum*? A viewpoint from the Netherlands. Avian Pathol. 2014;43(1):2–8. 24397240 10.1080/03079457.2014.881049

[16] Feberwee A, de Wit S, Dijkman R. Clinical expression, epidemiology, and monitoring of *Mycoplasma gallisepticum* and *Mycoplasma synoviae*: an update. Avian Pathol. 2022;51(1):2–18. 34142880 10.1080/03079457.2021.1944605

[17] El-Ashram S, Hashad ME, Abdel-Alim GA, Abdelhamid T, Deif N. Seroprevalence of mycoplasmosis in broiler, layer, and native chickens in Giza, Egypt. PLoS ONE. 2021;16(7):e0254220. 34252126 10.1371/journal.pone.0254220 PMC8274858

[18] Tipu JH, Miah R, Islam O, Rahman MM, Talukdar L, Miah R, Hussain MS, Islam MA, Ahsan MI, Raquib A, Noor M. Molecular and Seroprevalence of *Mycoplasma gallisepticum* in Turkeys in Sylhet district of Bangladesh. Vet Med Sci. 2025;11(2):e70227. 39999283 10.1002/vms3.70227 PMC11855371

[19] Ewing ML, Kleven SH, Brown MB. Comparison of enzyme-linked immunosorbent assay and hemagglutination-inhibition for detection of antibody to *Mycoplasma gallisepticum* in commercial broiler, fair and exhibition, and experimentally infected birds. Avian Dis. 1996;40(1):13–22. 8713026

[20] Hu ZJ, Li HR, Zhao YX, Wang GJ, Shang YB, Chen YT, et al. NADH oxidase of *Mycoplasma synoviae* is a potential diagnostic antigen, plasminogen/fibronectin binding protein and a putative adhesin. BMC Vet Res. 2022;18:455. 36581820 10.1186/s12917-022-03556-2 PMC9798693

[21] Razin S, Yogev D, Naot Y. Molecular biology and pathogenicity of Mycoplasmas. Microbiol Mol Biol Rev. 1998;62(4):1094–156. 9841667 10.1128/mmbr.62.4.1094-1156.1998 PMC98941

[22] Bencina D. Haemagglutinins of pathogenic avian Mycoplasmas. Avian Pathol. 2002;31(6):535–47. 12593736 10.1080/0307945021000024526

[23] Noormohammadi AH, Markham PF, Markham JF, Whithear KG, Browning GF. *Mycoplasma synoviae* surface protein MSPB as a Recombinant antigen in an indirect ELISA. Microbiol (Reading). 1999;145(8):2087–94. 10.1099/13500872-145-8-2087 10463175

[24] Noormohammadi AH, Browning GF, Jones J, Whithear KG. Improved detection of antibodies to *Mycoplasma synoviae* vaccine MS-H using an autologous Recombinant MSPB enzyme-linked immunosorbent assay. Avian Pathol. 2002;31(6):611–7. 12593746 10.1080/0307945021000024553

[25] Noormohammadi AH, Markham PF, Whithear KG, Walker ID, Gurevich VA, Ley DH, et al. *Mycoplasma synoviae* has two distinct phase-variable major membrane antigens, one of which is a putative hemagglutinin. Infect Immun. 1997;65(7):2542–7. 9199417 10.1128/iai.65.7.2542-2547.1997 PMC175359

[26] Noormohammadi AH, Markham PF, Kanci A, Whithear KG, Browning GF. A novel mechanism for control of antigenic variation in the haemagglutinin gene family of *Mycoplasma synoviae*. Mol Microbiol. 2000;35(4):911–23. 10692167 10.1046/j.1365-2958.2000.01766.x

[27] Yadav JP, Tomar P, Singh Y, Khurana SK. Insights on and infection in poultry: a systematic review. Anim Biotechnol. 2022;33(7):1711–20. 33840372 10.1080/10495398.2021.1908316

[28] Stipkovits L, Kempf I. Mycoplasmoses in poultry. Rev Sci Tech. 1996;15(4):1495–525. 9190023 10.20506/rst.15.4.986

[29] Sun SK, Lin X, Chen F, Wang DA, Lu JP, Qin JP, et al. Epidemiological investigation of *Mycoplasma Synoviae* in native chicken breeds in China. BMC Vet Res. 2017;13(1):115. 28441945 10.1186/s12917-017-1029-0 PMC5405555

[30] Sui C, Cui H, Ji J, Xu X, Kan Y, Yao L, et al. Epidemiological investigations and locally determined genotype diversity of *Mycoplasma synoviae* in central China from 2017 to 2019. Poult Sci. 2022;101(1):101522. 34818613 10.1016/j.psj.2021.101522 PMC8626675

[31] Levisohn S, Kleven SH. Avian mycoplasmosis (*Mycoplasma gallisepticum*). Rev Sci Tech. 2000;19(2):425–42. 10935272

[32] Cortes V, Sevilla-Navarro S, Garcia C, Tudon A, Marin C, Catala-Gregori P. Seroprevalence and prevalence of *Mycoplasma synoviae* in laying hens and broiler breeders in Spain. Poult Sci. 2021;100(3):100911. 33518326 10.1016/j.psj.2020.11.076 PMC7936174

[33] Moreira FA, Cardoso L, Coelho AC. Epidemiological survey on *Mycoplasma synoviae* infection in Portuguese broiler breeder flocks. Vet Ital. 2015;51(2):93–8. 26129659 10.12834/VetIt.116.329.3

[34] Christodoulides A, Gupta N, Yacoubian V, Maithel N, Parker J, Kelesidis T. The role of lipoproteins in mycoplasma-mediated Immunomodulation. Front Microbiol. 2018;9:1682. 30108558 10.3389/fmicb.2018.01682 PMC6080569

[35] Szczepanek SM, Frasca S, Schumacher VL, Liao X, Padula M, Djordjevic SP, et al. Identification of lipoprotein MslA as a neoteric virulence factor of. Infect Immun. 2010;78(8):3475–83. 20515935 10.1128/IAI.00154-10 PMC2916287

[36] Han SZ, Wang Y, Wang LZ, Chang WC, Wen B, Fang JY et al. LP78 is a fibronectin/plasminogen binding protein, putative adhesion, and potential diagnostic antigen. Front Microbiol. 2024;14:1335658. 10.3389/fmicb.2023.1335658 PMC10803467 38264482

[37] Wang Y, Li H, Wen Z, Shang YB, Liu T, Ding C, et al. Establishment and application of ELISA method for detection of *Mycoplasma synoviae* antibody based on lipoprotein P80. Acta Microbiol Sinica. 2020;60(3):512–24.

[38] Qi J, Wang Y, Li H, Shang Y, Gao S, Ding C, et al. *Mycoplasma synoviae* Dihydrolipoamide dehydrogenase is an Immunogenic fibronectin/plasminogen binding protein and a putative adhesin. Vet Microbiol. 2022;265:109328. 35032790 10.1016/j.vetmic.2021.109328

[39] Simionatto S, Marchioro SB, Galli V, Luerce TD, Hartwig DD, Moreira AN, Dellagostin OA. Efficient site-directed mutagenesis using an overlap extension-PCR method for expressing *Mycoplasma hyopneumoniae* genes in *Escherichia coli*. J Microbiol Methods. 2009;79(1):101–5. 19733599 10.1016/j.mimet.2009.08.016

[40] Chen R, Shang H, Niu X, Huang J, Miao Y, Sha Z, et al. Establishment and evaluation of an indirect ELISA for detection of antibodies to goat Klebsiella pneumonia. BMC Vet Res. 2021;17(1):107. 33663505 10.1186/s12917-021-02820-1 PMC7934495

[41] Zou KH, O’Malley AJ, Mauri L. Receiver-operating characteristic analysis for evaluating diagnostic tests and predictive models. Circulation. 2007;115(5):654–7. 17283280 10.1161/CIRCULATIONAHA.105.594929

[42] Xu B, So WKW, Choi KC. Determination of a cut-off comprehensive score for financial toxicity (COST) for identifying cost-related treatment nonadherence and impaired health-related quality of life among Chinese patients with cancer. Support Care Cancer. 2024;32(2):136. 38279988 10.1007/s00520-024-08320-w PMC10821980

