# Supplementary material for: Development of an indirect ELISA based on a new specific lipoprotein LP53 for the detection of antibodies against Mycoplasma synoviae
Source: BMC Vet Res. 2025 May 31;21:393. doi: 10.1186/s12917-025-04827-4 (PMC12125734; doi:10.1186/s12917-025-04827-4)
Supplement: Supplementary file 1 — Supplementary Material 1 [file 12917_2025_4827_MOESM1_ESM.docx]

**Supplementary figures**

**Fig. S1**

**A**

M 1 2 3 4 5 M 6







M 1

M 1 2 3

**B**

**C**




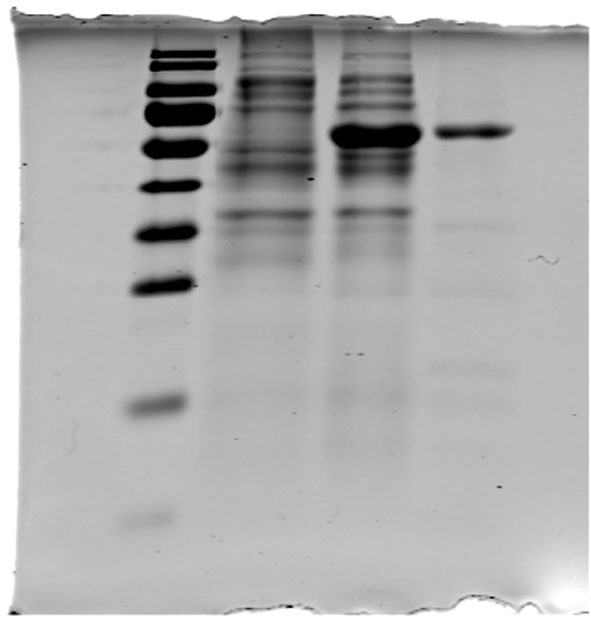


**Fig. S1 Original figures for Fig.1 (Amplification, recombinant plasmid construction, expression and purification of MSLP53)**. (A) Overlap PCR products for MS*lp53* gene. M: DL2 000 DNA marker; 1-5: PCR product with primers MS*lp53* 1F/1R-5F/5R; 6: full length of MS*lp53* gene produced by overlap PCR amplification. (B) Double enzyme digestion of recombinant expression plasmid. M: DL5 000 DNA marker; 1: the recombinant expression plasmid pCold-MS*lp53* digested by *Bam*H I/*Eco*R I. (C) Expression and purification of recombinant MSLP53 protein. 1: IPTG-induced *E. coli* BL21 cells containing empty pCold I vector; 2: supernatants from IPTG induced *E. coli* BL21 containing pCold I-MS*lp53*; 3: purified rMSLP53 protein.

**Fig. S2**

Lane 1 Lane 2 Lane 3 Lane 4 Lane 5 Lane 6


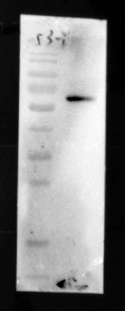

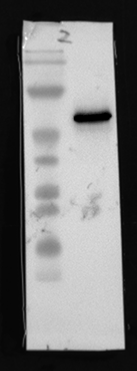

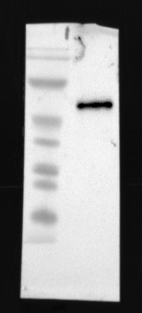

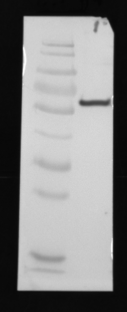

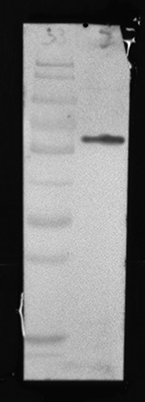

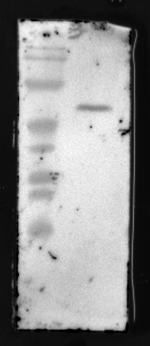


Standard MS(+)

MS JS1 (+)

MS HB1 (+)

MS SH1 (+)

MS SD1 (+)

MS WVU_1853_ (+)

Lane 7 Lane 8 Lane 9 Lane 10 Lane11 Lane 12 Lane 13


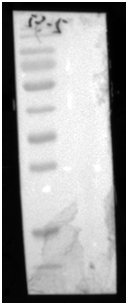

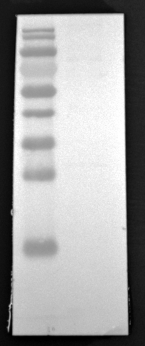

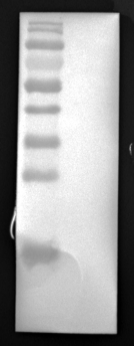

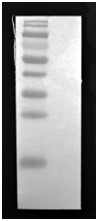

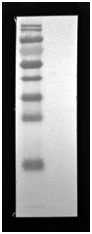

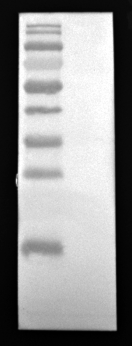

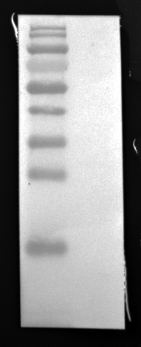


MG SS (+)

MG SGN (+)

MG FBH (+)

MG 08 (+)

MG 013 (+)

MG Rlow (+)

Standard MG(+)

Lane 14 Lane 15 Lane 16 Lane 17 Lane18 Lane 19 Lane 20


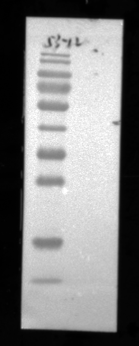

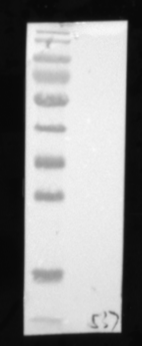

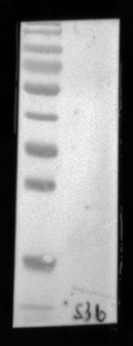

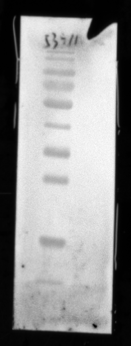

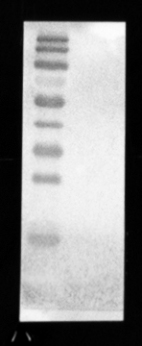

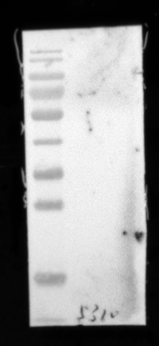

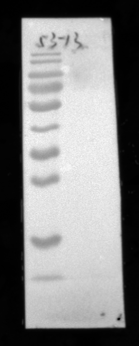


MS (-)

IBDV (+)

IBV (+)

NDV (+)

*E.coli* (+)

SPG (+)

MI (+)

**Fig. S2 Original figures for Fig.2 (Immunoreactivity and specificity analysis of rMSLP53 with different chicken sera).** The purified rMSLP53 protein was used as antigen to react with standard MS-positive chicken serum (standard MS+) and chicken positive sera of against different MS isolates (MS WVU_1853_, JS1, SD1, SH1 and HB1); MG-positive chicken sera (standard MG+) and different MG-positive chicken sera (MG Rlow, 013, 08, FBH, SGN, SS); positive sera against other avian pathogens (MI, SPG, *E. coli* O1/O2/O78, NDV, IBV and IBDV) and MS-negative serum.
